# Patient, family and provider views of measurement-based care in an early-psychosis intervention programme

**DOI:** 10.1192/bjo.2021.1005

**Published:** 2021-09-17

**Authors:** Ari B. Cuperfain, Katrina Hui, Suze G. Berkhout, George Foussias, David Gratzer, Sean A. Kidd, Nicole Kozloff, Paul Kurdyak, Brandon Linaksita, Dielle Miranda, Sophie Soklaridis, Aristotle N. Voineskos, Juveria Zaheer

**Affiliations:** Department of Psychiatry, University of Toronto, Canada; Department of Psychiatry, University of Toronto, Canada; and University Health Network, Canada; Centre for Addiction and Mental Health, Canada; Department of Psychiatry, University of Toronto, Canada; Centre for Addiction and Mental Health, Canada; Centre for Addiction and Mental Health, Canada; and Mental Health and Addictions Research Program, Institute for Clinical Evaluative Science (ICES), Canada; Centre for Addiction and Mental Health, Canada; Department of Psychiatry, University of Toronto, Ontario; Centre for Addiction and Mental Health, Canada; and Department of Family and Community Medicine, University of Toronto, Canada; Department of Psychiatry, University of Toronto, Canada; and Centre for Addiction and Mental Health, Canada; Department of Psychiatry, University of Toronto, Canada; Centre for Addiction and Mental Health, Canada; and Health Outcomes and Performance Evaluation (HOPE) Research Unit, Institute for Mental Health Policy Research Canada

**Keywords:** Measurement-based care (MBC), psychosis, interprofessional team, transitional youth, qualitative

## Abstract

**Background:**

Measurement-based care (MBC) in mental health improves patient outcomes and is a component of many national guidelines for mental healthcare delivery. Nevertheless, MBC is not routinely integrated into clinical practice. Several known reasons for the lack of integration exist but one lesser explored variable is the subjective perspectives of providers and patients about MBC. Such perspectives are critical to understand facilitators and barriers to improve the integration of MBC into routine clinical practice.

**Aims:**

This study aimed to uncover the perspectives of various stakeholders towards MBC within a single treatment centre.

**Method:**

Researchers conducted qualitative semi-structured interviews with patients (*n* = 15), family members (*n* = 7), case managers (*n* = 8) and psychiatrists (*n* = 6) engaged in an early-psychosis intervention programme. Data were analysed using thematic analysis, informed by critical realist theory.

**Results:**

Analysis converged on several themes. These include (a) implicit negative assumptions; (b) relevance and utility to practice; (c) equity versus flexibility; and (d) shared decision-making. Providers assumed patients’ perspectives of MBC were negative. Patients’ perspectives of MBC were actually favourable, particularly if MBC was used as an instrument to engage patients in shared decision-making and communication rather than as a dogmatic and rigid clinical decision tool.

**Conclusions:**

This qualitative study presents the views of various stakeholders towards MBC, providing an in-depth examination of the barriers and facilitators to MBC through qualitative investigation. The findings from this study should be used to address the challenges organisations have experienced in implementing MBC.

## Background

**Fig. 1:**
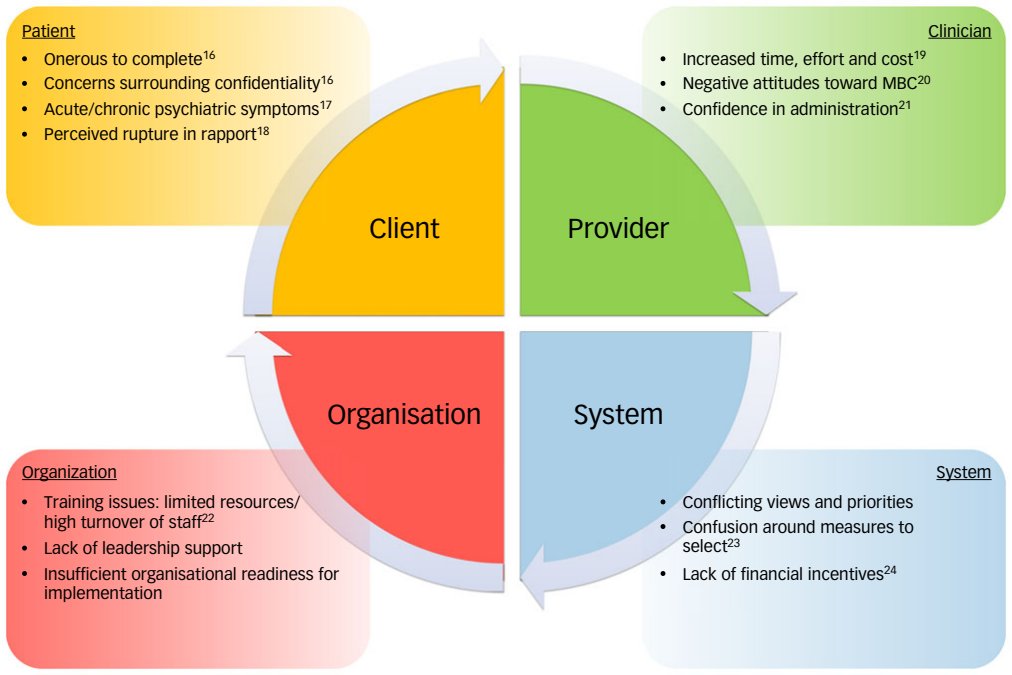
Barriers to implementing measurement-based care (MBC) in routine clinical practice. Barriers can be categorised at the level of the patient, the clinician, the organisation and the system.^15^

Measurement-based care (MBC) shows potential as a crucial component of psychiatric service delivery.^1^ MBC relies on the administration of symptom rating scales to record and track meaningful patient outcomes for use in standardised clinical decision-making.^2,3^ Empirically monitoring patient progress is considered evidence-based standard of care in mental health guidelines.^4^ Indeed, patient outcomes are improved in certain studies with the utilisation of MBC compared with standard care as shown in depression,^5,6^ eating disorders,^7^ substance use^8^ and other conditions. In addition, MBC is effective in diverse clinical populations including youth,^9,10^ couples^11^ and soldiers.^12^ However, these results are not uniform across all studies, perhaps because of the heterogeneity of such programmes,^13^ and factors related to implementation and sustainability of the specific MBC framework.^14^ Although MBC is gaining acceptance in mental health, it still remains underused and carries systemic barriers to successful widespread implementation (Fig. 1), as recently reviewed, with less than 20% of providers integrating MBC into their practice.^15^

Overall, the view of care providers towards MBC is mixed.^25,26^ Regardless of the positive or negative viewpoints expressed, providers rarely incorporate it regularly into practice.^27^ In addition to providers, the perspective of patients towards MBC has an impact on successful uptake of MBC. A synthesis of past qualitative studies concluded that MBC has potential to be accepted by patients if it is perceived as for their benefit and empowers them in shared decision-making.^28^ Indeed, whereas providers often fear that using MBC will disrupt the therapeutic relationship, MBC can actually enhance the therapeutic relationship.^29^

The Slaight Centre Early Intervention Services (SCEIS) is a coordinated specialty care programme located in Toronto, Canada, offered to young people aged 16–29 experiencing psychosis. Patients are followed for up to 3 years and interact with psychiatrists, case managers and peer-support workers through the programme. A move towards MBC was introduced in the context of a broader integrated care pathway in June 2015 to provide standardised early-psychosis intervention (EPI) care. Scheduled measures of symptoms, functioning and side-effects were collected and routinely completed for each patient (Appendix 1). Some of these measurements were selected by the programme and others were mandated by the hospital. Over the course of 13 months, average completion rates were 31%, with variability between clinicians and psychiatrists, and it was not clear that the results were being used to inform clinical care, including monthly symptom and improvement ratings that could be well-suited for this purpose. For example, the Clinical Global Impression (CGI)^30^ and Brief Psychiatric Rating Scale (BPRS)^31^ were conducted monthly with the goal of reviewing changes in these measurements during weekly team meetings to identify improvement or worsening in symptoms, however, this was inconsistently applied in practice. Despite providing training and support for implementing MBC, decision-makers perceived resistance from providers, and sought to better understand the attitudes of patients and families as well as providers in the programme about the new model. Although barriers to effective implementation of MBC have been identified in mental health services broadly, little has been written of implementation and uptake of MBC in EPI programmes.

## Aims

Our study aimed to characterise the perspectives of patients, families and care providers of a recently implemented MBC initiative at an EPI service, using qualitative analysis informed by critical realist theory. Critical realism is particularly useful in a healthcare programme evaluation because it can bridge traditional measures of programme effectiveness with qualitative enquiry to uncover complex social and structural factors.^32^ This work adds important context to the question of factors that influence acceptance of MBC. To our knowledge, ours is the first to assess patient, family, case worker and psychiatrist experiences simultaneously within the same programme. As well, the population of patients receiving EPI represents a group likely to benefit from MBC, yet with unique challenges for promoting engagement and development of a therapeutic relationship.

## Method

### Research design

The qualitative interviews and analysis in the study were informed by critical realist theory. Critical realist theory suggests that the structure of a programme is shaped by people at various levels of power within it, and acceptance of or resistance to pieces of the intervention can lead to issues and challenges in programme delivery that have unintended positive or negative consequences.^33^ Qualitative analysis was conducted using the principles of thematic analysis, which has been frequently used in qualitative research in mental health in general and first-episode psychosis in particular.^34–37^

### Ethics

Informed written and verbal consent was obtained from participants prior to their involvement in this study. The authors assert that all procedures contributing to this work comply with the ethical standards of the relevant national and institutional committees on human experimentation and with the Helsinki Declaration of 1975, as revised in 2008. All procedures involving human participants were approved by the Centre for Addiction and Mental Health, REB 140/2016.

### Participants

Participants for this study included patients, family members and care providers. Patients were 16 years of age or older, had been seen in consultation at the SCEIS programme, and had been followed for at least 3 months. They were ineligible if they could not speak English fluently, were currently in hospital or had a history of neurological impairment, or significant visual/auditory impairment. ‘Family’ were caregivers or loved ones of patients and had also attended at least part of a meeting at SCEIS with the patient during their care. They were excluded only if they could not speak English fluently.

All physicians or case managers working with SCEIS were eligible, and all were approached to participate. Given the narrow eligibility criteria, patients and families were consecutively approached by research team members and provided information about the study. Sample size was guided by achieving sufficient information power; given that recruitment ended prior to analysis, saturation was not explicitly used to guide sample size, although no new themes emerged towards the end of analysis.^38^

### Interviews

The development of the interview guide was informed by five ‘stakeholder engagement’ discussions that were not taped or transcribed. In-person interviews were then conducted between May 2017 to August 2017 by J.Z. and B.L. Participants provided basic demographic and diagnostic information as applicable. Questions focused on thoughts around the use of scales, implementation of MBC and factors associated with engagement in the EPI programme (see Interview Guides in Supplementary Appendix 1 available at https://doi.org/10.1192/bjo.2021.1005); although not initially intended to focus solely on MBC, rich responses throughout the interviews focused on this important aspect of the programme.

Participants were informed that the authors had experience in qualitative research and had been asked to conduct a programme evaluation study. They were given the opportunity to book a second interview if they wished to add more information, but no participants requested this. Memos were written following the interviews and entered into NVivo. Full interview transcripts were seen only by J.Z., B.L., A.B.C. and K.H.

### Data analysis

Interview audio files were professionally transcribed by a third-party with no relationship with SCEIS, and participants were informed of this fact. Transcripts were reviewed for accuracy and then uploaded into NVivo for coding. Transcripts were read and coded independently by three researchers (A.B.C., K.H., J.Z.), and a reflexivity journal was recorded throughout. Once coding was complete, the researchers used thematic analysis to arrive at themes.^39^ As in all variants of thematic analysis, six steps were undertaken: (a) familiarising oneself with the data through close reading, (b) generating initial codes, (c) searching for themes, (d) reviewing themes (e) defining themes, and (f) writing up the findings.^39^ Results were compared, differences were discussed and final themes were reached by consensus. Overall, the study adhered to COREQ^40^ and SRQR^41^ guidelines for qualitative research.

### Reflexivity statement

Our authorship team consisted of health professionals, researchers and administrators, with a variety of clinical and research experience and backgrounds. Some worked within SCEIS whereas others did not, allowing for an informed, balanced and unbiased approach. The interviews and transcript analysis were conducted by investigators at an arm's length from the SCEIS, who have past experience in understanding and investigating patient experiences in the mental health system. Reflexivity statements for specific authors can be found in Supplementary Appendix 2.

## Results

### Results overview

We conducted interviews with patients (*n* = 15), family members (*n* = 7), case managers (*n* = 8) and psychiatrists (*n* = 6). Demographic data of the interview participants are presented in Table 1. Overall, we found that many care providers had negative perceptions pertaining to certain aspects of MBC, yet they agreed with the overall mandate of ensuring consistency of care across the programme, empirical monitoring of symptoms and evidence-based care. They feared that implementing MBC would be overly time-consuming and risked damaging the patient–provider therapeutic relationship.

**Table 1:** Summary statistics of patients, family and care provider participants

Participants	Values
*Patients (n = 15)*	
Demographics	
Female, *n*	4
Male, *n*	8
Not recorded	3
White, % (*n*)	47 (7)
Age, mean (s.d.)	25.7 (4.8)
Diagnosis	
Schizhophrenia/schizoaffective	7
Bipolar disorder	5
Other	3
*Family (n = 7)*	
Demographics	
Female	6
Male	1
White, % (*n*)	71 (5)
Age, mean (s.d.)	49.0 (16.9)
Diagnosis of patient	
Schizhophrenia/schizoaffective	2
Bipolar disorder	2
Other	3
*Care providers (n = 14)*	
Demographics	
White, % (*n*)	43 (6)
Age, mean (s.d.)	41.7 (7.7)
Case managers, % (*n*)	57 (8)
Years experience, *n*	
Less than 1	1
1–5	6
5–9	5
≥10	2

Paradoxically, patients and family viewed MBC more positively provided that it did not detract from the relational parts of care and rather facilitated communication and shared decision-making. Specific themes that emerged from the data are detailed below, along with selected sample quotations (Appendix 2; see also Supplementary Table 1 for additional quotations).

### Theme 1: implicit negative assumptions

Three subthemes emerged related to implicit perspectives of MBC – patients’ perspectives of MBC, providers’ perspective of MBC and care providers’ assumptions about patients’ perspectives on MBC. There was significant divergence between care providers’ assumptions about patients’ perspectives of MBC and patients’ self-report of MBC perspectives.

The majority of care providers perceived that MBC was experienced negatively by patients. Specifically, they identified that scales were onerous to complete and had a negative impact on the therapeutic relationship, potentially threatening service engagement. However, despite the perceived negative views of care providers, patient interviews indicate an almost universally positive view about MBC. When asked about cognitive assessment scales, one patient responded:

‘I love them, yeah, I really like them.’ (Patient (P)11)

Others were less enthusiastic, yet still felt the benefits outweighed the inconvenience.

Families were often more concerned about the implications of MBC in their ability to maintain psychiatric resources. Specifically, they feared that with the additional time requirements of conducting and administrating MBC, coupled with the manualised process of illness severity monitoring, patients would be managed increasingly by primary care physicians, rather than psychiatrists.

In addition to the perceptions of care providers towards patients’ acceptance of MBC, themes around their own acceptance of MBC emerged. Care providers felt that MBC was time-consuming and added additional tasks to their already busy schedules. There was also a feeling of frustration that the implementation of the MBC had been top-down, without full appreciation of the consequences for care providers. As one case manager noted, introducing MBC was:

‘very top down, [an] absolutely top-down process with no input [from] front line staff.’ (Case manager (c)10)

### Theme 2: relevance and utility to practice

Another theme that emerged was a feeling that at least certain aspects of MBC were irrelevant, do not appropriately capture patient details, and do not contribute to overall care. Every case manager indicated that, in general, psychiatrists do not look at the measures they complete, and, in turn, case managers do not review the psychiatrists’ measures. Many attributed this to the selection of particular scales that were either irrelevant or redundant. Notwithstanding, many still felt that there was value in MBC for detecting and monitoring patient symptoms.

This sentiment was echoed by psychiatrists as well. They noted the benefits of routine monitoring and evidence-based care. However, they also expressed reservations that the current tools may be oversimplified or irrelevant for certain patients.

Patients and their families acknowledged the utility of MBC in establishing a baseline, detecting symptoms early and using results to guide treatment decisions. Most patients described ways in which scales are helpful in their care, for example:

‘I think that it's good to have [MBC] to maybe get like a vague gauge of how the patient is doing at that time…yeah, another point of reference, you know.’ (P11)

Interestingly, two separate family members likened MBC for EPI to their own healthcare experience such as symptom management in oncology.

### Theme 3: equity versus flexibility

Many care providers identified equity as a significant benefit of MBC. The implementation of MBC necessitates standardisation of care by requiring the care provider to consider and assess various domains of symptoms and function. By standardising care, resources were offered to patients routinely, eliminating the need for subjective judgement and reducing the likelihood of bias. At the same time, care providers feared that MBC could threaten their ability to adapt treatment to an individual. Flexibility was identified as an essential component of fostering a therapeutic alliance and promoting engagement with the programme.

Patients and family members described this tension, as well. On the one hand, MBC was seen as more equitable, yet it also risked minimising the importance of the individual, not accounting for the patient's ability (or inability) to engage and being too rigid. While discussing an MBC-informed medication algorithm, one patient explained:

‘it would have to be pretty flexible because everybody reacts differently to different drugs.’ (P14)

With respect to standardisation, one mother said she:

‘wouldn't want to take it too far so that it's not taking into account [the] individual.’ (Family (F)02)

Another patient's partner felt that checklists and questionnaires should be used as an additional data point but not the:

‘be all and end all.’ (F01)

### Theme 4: shared decision-making

The last theme related to establishing the patient as an active force in any management plans deriving from MBC. Incorporating MBC had the opportunity to empower patients in their care but also carried the risk of excluding them from the process. Many care providers described using MBC to engage patients in their care, and to promote greater insight into the trajectory of their symptoms.

The futility of MBC in the absence of shared decision-making was epitomised by one patient who confided that he alters his responses because of fears it will affect his treatment at the expense of his own agency. This patient was fearful that his medication regimen would change strictly based on a numeric score on a monitoring scale, without incorporating his wishes and values. With respect to responding to scales as part of MBC, he stated that:

‘sometimes it is always that feeling [of] can I truthfully be honest, because I know if I say certain things, they will take something away or try to add something and I am comfortable already.’ (P01)

The other aspect related to shared decision-making that emerged was the power of MBC in facilitating communication. From the patient–provider dyad, MBC helped patients find words to focus on their experience:

‘You can narrow down and you can really recognise the way that you are feeling.’ (P08)

In addition to enhancing communication between the provider and the patient, MBC fosters interdisciplinary communication between case managers and psychiatrists co-managing a patient.

## Discussion

Numerous barriers exist in implementing MBC, with factors broadly categorised at the level of the patient, the provider, the organisation and the system. Certain barriers are common to all areas of medicine and healthcare, while others are specific to mental health. The response burden on patients in completing scales may be heightened. Importantly, in patients experiencing symptoms such as mania and psychosis there may be concerns around relevance to care, confidentiality and difficulty in completing scales at every visit. These concerns can extend towards the treatment team, limiting the utility of MBC in certain populations. Nevertheless, for people experiencing psychosis, MBC can be extraordinarily effective in balancing biological, psychological and social interventions to provide the highest quality of care.

### Main findings

**Fig. 2:**
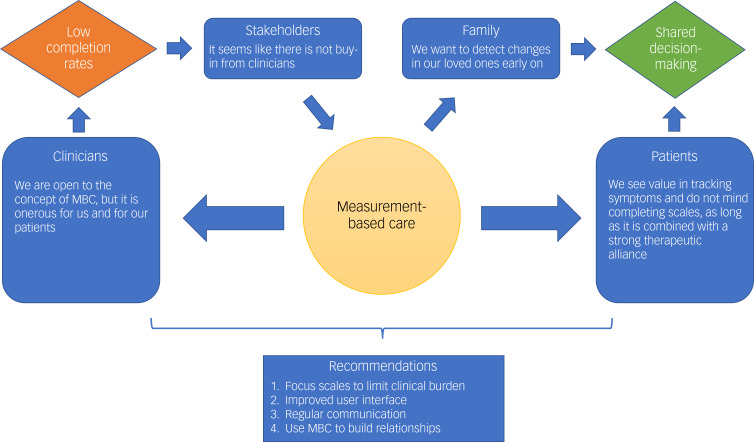
Schematic representation outlining the perspectives and relationships of patients, family, care providers and stakeholders towards implementing and operationalising measurement-based care (MBC) in an early-psychosis intervention (EPI) population. Although they agreed with the basic mandate of MBC, clinicians felt its implementation was overly top-down, and that the scales were too time-consuming for both them and their patients. This led to low response rates. However, patients and their families resonated with the idea of objective measurements, but not at the expense of flexibility and the individualistic part of care. MBC worked well when it empowered patients as an agent in their care through shared decision-making.

We sought the perspectives of patients, family, case managers and psychiatrists on MBC within an EPI programme. In short, perceived top-down administration of the programme resulted in push back, which led decision-makers to think that providers rejected MBC. Providers wanted to monitor patients empirically, but felt there was insufficient time, that the scales were not analysed and thus irrelevant, that MBC would damage the relationship with the patient and that the patient would have a negative experience using measurement tools. However, patients and their families actually resonated with the idea of objective measurements, but not at the expense of flexibility and the individualistic part of care. Instead, MBC worked well when it empowered them as an agent in their care through shared decision-making. A graphical summary of these findings is presented in Fig. 2.

### Comparison with findings from other studies

To our knowledge, our study was the first to incorporate multiple participant viewpoints towards MBC in interview format within the same psychiatric services programme, specifically in EPI. Other qualitative studies exploring this topic have focused more narrowly on participants,^42–46^ or used other modes of data collection (for example surveys, focus groups).^47–49^ As such, our findings can be used to understand conflicting results surrounding the efficacy of MBC more generally, allowing for triangulation of generated themes. Overall, our study provides crucial perspective by soliciting the viewpoints of multiple stakeholders within a specific programme utilising MBC, not otherwise captured from single-group or multiprogramme analysis.

Through a critical realist lens, we uncovered perceptions about MBC that would otherwise be difficult to measure. MBC is a technology that is both embedded in, and demonstrative of, social relations in clinical settings as much as it is an empirical decision-support and standardisation of care tool. Accordingly, in exploring the viewpoints of patients, families, providers and stakeholders, we were able to explore the relational aspects of implementing MBC within an integrated mental health service model.

The selection of scales used in the studied EPI programme included a combination of clinician-generated and patient-reported measures. Patient-reported outcome measurement scales were only completed annually, and this may explain the perceived burden of completing scales as a projection onto patients of their own increased burden. Moreover, this also could have influenced the perception of questionable utility endorsed by several clinicians in part because it did not include any patient-reported outcome measures completed frequently enough to guide clinical decision-making. It is notable that this same programme adopted the NAVIGATE model of care that includes a regularly completed patient-reported outcome measure that evaluates symptoms and side-effects.^50^ Indeed, in thinking about selection of scales, they should be meaningful to patients and families, feasible, quick, validated and able to guide shared decision-making.

### Limitations

Our study has several limitations. First, our study was based in one specialised programme with a specific patient demographic. Although the single-programme analysis was also a strength of this study, given the heterogeneity of MBC, our findings may not generalise to other settings, or to scales not used in our study. Second, although in retrospect patients did not feel that scales were onerous, and they were able to rationalise the importance of such scales, these perceptions may have differed at the time of assessment, which could account for the discrepancy between provider and patient perspectives in our study. It is also possible that there was selection bias in that those who were more likely to participate in these interviews would also have been more amenable to participating in MBC. In addition, despite discussion around confidentiality and anonymity, participants may have altered their responses for fear of negatively have an impact on their care or careers. However, the range of responses and candid feedback suggests they were comfortable providing candid accounts of their experiences. Notwithstanding, acceptance of new programmes including MBC is variable with individual factors contributing in ways not completely captured in this study.

### Implications

The participant interviews converge on a number of recommendations for developing and implementing successful MBC programmes. First, specific measurement scales should be carefully selected, with clear advanced guidelines and training on how to use these tools. Second, there should be regular and ongoing communication between team members about the data. Lastly, MBC should be used to enhance the relationship with patients and foster shared decision-making.

This study explored the various factors that may facilitate or impede successful implementation of MBC in mental healthcare delivery through a much-needed qualitative framework. Our data showed that care providers want to provide a high-quality, evidence-based practice, without losing sight of the relational aspects of care. Balancing the importance of evidence-based, measurement-informed care with interventions and modalities that have meaning for patients and families is crucial not only in promoting the engagement of those using services, but also of those providing care. Addressing barriers and challenges iteratively while incorporating the viewpoints of all involved may help improve the efficacy of current MBC protocols, and guide development of future programmes. Keeping in mind the relational aspects of MBC in practice may improve its overall integration towards informing patient care.

## Data Availability

The data that support the findings of this study are available from the corresponding author, J.Z., upon reasonable request.

## Supplementary material

For supplementary material accompanying this paper visit http://dx.doi.org/10.1192/bjo.2021.1005. click here to view supplementary material

## Appendices

**Appendix 1:** Summary of scales used as part of measurement-based care in the Slaight Centre Early Intervention Services early-psychosis intervention programme

Assessment	Construct	Who completes	Frequency
Brief Psychiatric Rating Scale (BPRS)^31^	Overall psychopathology and psychotic symptom severity	Physician	Every 4 weeks until response criteria met, then 3 months, 6 months, annually
Clinical Global Impression (CGI)^32^	Overall illness severity and improvement	Physician	Every 4 weeks until response criteria met, then 3 months, 6 months, annually
Birchwood Social Functioning Scale^51^	Social functioning	Patient	Annually
Brief Cognitive Assessment Tool for Schizophrenia (BCATS)^52^	Neurocognitive symptoms	Case manager	Annually
Personal and Social Performance Scale (PSP)^53^	Functional status	Case manager	Annually
Quality of Life Enjoyment and Satisfaction Questionnaire (Q-LES-Q)^54^	Subjective quality of life	Patient	Annually
Barnes Akathisia Rating Scale (BARS)^55^	Medication side-effects: akathisia	Physician	Annually
Abnormal Involuntary Movement Scale (AIMS)^56^	Medication side-effects: dyskinesias	Physician	Annually
Simpson-Angus Scale (SAS)^57^	Medication side-effects: parkinsonism	Physician	Annually

**Appendix 2:** Summary of results from qualitative analysis

Theme	Quotations
Theme 1: implicit negative assumptions	‘Oh I love those [scales]… Yeah, I love them, yeah, I really like them.’ (P11) ‘It is annoying sometimes, but then I realise that there might be a reason why they are doing it.’ (P13) ‘You need to make sure that you have the resources to [implement MBC], to handle that, because the push right now from what I've experienced is that they will push you to your family doctor.’ (F03) ‘Some of them just don't want to sit down with their case managers and do the tests and the questionnaires.’ (S01) ‘When you sit down some of these clients are alienated by these type of batteries.’ (C06) ‘Some patients don't like to hear the same questions over and over and over.’ (S13)
Theme 2: relevance and utility to practice	‘I feel like it is not really going to change [outcomes], but it is not going to annoy me, because I mean you have to develop an average somehow.’(P03) ‘The vast majority of people gravitate towards quantitative information so I think it is helpful.’ (P15) ‘The issue is you can't measure what you can't see…I think, most parents want to do what is right and will only want to do what's right in a measurable way.’ (F04) ‘I'd get an appreciation of where people are at from baseline to where they are at different times throughout their treatment.’ (C09) ‘We entered [the scores] and then it kind of goes into the system and disappears.’ (C09)
Theme 3: equity versus flexibility	‘Being able to offer the same thing to everybody I thought was [a] really great idea. Being able to be more evidence based, more like looking at more functional outcomes I thought that was [positive].’ (C07) ‘People decline and you respectfully appreciate that. I think it can be used to build rapport only from a, “I want to get to know you better, and these are some tools that I can do that” [standpoint].’ (C02)
Theme 4: shared decision-making	‘The only way that I think that it could help would be like look here is a 5, and then [case manager's name] would be like why do you feel that it is a 5?’ (P08) ‘[MBC] can be helpful like if someone doesn't feel comfortable talking about something, I guess it can help them get a better understanding.’ (P10) ‘It's a way of communicating with other team members.’ (S01)

C, case manager; F, family; MCB, measurement-based care; P, patient; S, psychiatrist.
