# Supplementary material for: Patient, family and provider views of measurement-based care in an early-psychosis intervention programme
Source: BJPsych Open. 2021 Sep 17;7(5):e171. doi: 10.1192/bjo.2021.1005 (PMC8485347; doi:10.1192/bjo.2021.1005)
Supplement: Supplementary file 1 [file bjosup.zip › S205647242101005Xsup001.docx]

**Supplementary Table 1** – Additional quotations coded by theme

| **Theme** | **Quotations** |
| --- | --- |
| Theme 1: Implicit negative assumptions | Patients  [The cognitive test] was fun, it’s actually fun. Yeah it was fun I said I like those games yeah (P02).  I feel like it is not really going to change [outcomes], but it is not going to annoy me, because I mean you have to develop an average somehow (P03).  It was overwhelming. It was kind of a bit much, it was too many I remember (P05).  I don’t think it would be annoying (P06).  We just found it really tedious… Just not every single appointment, like if it is every 10^th^ appointment or every 6^th^ (P08).  [The cognitive test is] way too long. It’s way too long. I actually started hallucinating because of how long it was…[And with regards to symptom tracking], Oh, the same types of questions – good. I like that (P09).  Oh I love those [scales]… Yeah, I love them, yeah, I really like them (P11).  It is annoying sometimes, but then I realize that there might be a reason why they are doing it (P13).  Well, I think that it is important for consistency , just like from like a realistic perspective, it‘s important to be consistent… If you are getting frustrated with the question, then maybe you are having a bad day, because people are trying to help you here (P15).  Families  You need to make sure that you have the resources to [implement MBC], to handle that, because the push right now from what I’ve experienced is that they will push you to your family doctor (F03).  It sounds like [MBC] would be useful in terms of measurement over time, but, at the same time, like, if you saw [my daughter] now it would seem silly to ask her if she feel, ever feels paranoid just 'cause, like, it’s so far from how she was (F07).  Providers  Some of them just don’t want to sit down with their case managers and do the tests and the questionnaires (S01).  I do hear a lot of grumblings. I think they feel busy enough, and then having to do these various assessments it’s just more demands on their time. I think the way we, the psychiatrists have been trying to work with case managers around this is trying to normalize it as part of their assessments of clients anyways or just doing it in a different kind of way more standardized way (S01).  People then also perceived it as something that we are imposing on people and I think there was a large hiccup around maybe people’s choice of language and whether that was reflective of where they came from like oh geez this is stupid I hate doing this and that would obviously come across in how it’s addressed to clients as opposed to when saying like you know just getting a more wholesome picture of what’s going on and can really identify areas that maybe we can work on (C02).  Well I think there is a lot of resistance and some is not full understanding of what’s an ICP what’s going to be in our ICP and how it actually is going to provide better care and it doesn’t have to be more work and it’s the perception of more work like it’s all those pieces that come with change… The surprise is how much resistance there has been. A surprise is how difficult it has been for people to figure out how to weave in even the baseline measures and that being a huge resistance (C02).  Before I was feeling a lot of pressure oh you gotta do all this plethora of assessments and that was super imposed (C06).  Some people didn’t show up, and I suspect they didn’t show up because listen I told them listen okay you know I have these scales to do it will probably take me an hour an hour and a half to do can you come in we’ll get them all (C06).  When you sit down some of these clients are alienated by these type of batteries …and some clients didn’t want to get involved and you can see that some clients are somewhat alienated by it (C06).  I think if clients are in the space or are have the kind of personality that respond well to kind of algorithms and measurements and things like that then I think it can be helpful and it would be kind of like if this was going to help me then sure but I think that and it kind of depends on the scale too so something like metabolic monitoring and comfort and safety planning are completely different right so I think that most would probably be amenable (C07).  It would negatively impact care if you are spending too much time doing the scales and not speaking to the patient (S08).  In the beginning it was hard to kind of especially in the beginning you know you are trying to develop that rapport and that engagement with someone and you know I personally keep all my ICP stuff in a red binder and it’s alphabetical and I keep the sheets in there because I keep adding to them and what not and stopped bringing the red binder to appointments because it looked a little daunting so I just bring my ICP sheet and just explain to someone that you know for the first 3 months we are going to be going through a lot of these scales and what not just to get an understanding of base line and where people are at (C09).  Everybody hates [the OCAN], it’s a nightmare, nobody uses it, and then you do it and catch up (C11).  It's not something that [patients] express that they adore doing nor is it something that they get really distressed about…There's, there's a certain, I think, comfort, particularly with family members, that they're objective measures (S12).  I guess depending on the scales, like, if it's something that, you know, they need to be questioned about. Like, some patients don’t like to hear the same questions over and over and over (S13). |
| Theme 2: Relevance and utility to practice | Patients  I don’t know how helpful [scales] would be though? I think for someone maybe who has worse symptoms or is in a bit worse state, they might be helpful, you know just to see how they were interacting with the world, I guess (P06).  I feel like it is a good test for your brain and keeps you consist, like the questions especially like make sure that you don’t end up … like if you are experiencing manic symptoms you are treating them right away (P07).  if you do the test multiple times you would get better at taking it. So there is a learning curve involved. Like once I learned that I had to remember 3 back, like I could remember them by fingers, just like A is on this finger, C is on this finger, like once you get to know some tricks to learn them, it is a lot easier so it is not exactly standardized (P09).  And sometimes it is like I don’t constantly think about am I experiencing suicidal thoughts as much as I am experiencing suicidal thoughts so it sometimes nice to be cathartic and let my feelings out around those sort of things come out – it is very nice actually, so even bringing it up it can be a trigger for bad mood, but I like the Catharism of it (P09).  The vast majority of people gravitate towards quantitative information so I think it is helpful (P15).  Families  I think that they are certainly helpful. Ah … but they are not the be all and end all (F01).  [MBC] falls in line with what I’ve seen now at [name redacted] Hospital, when I go to see my Oncologist…when you check in and give them your card and everything, you have to answer a bunch of questions and it spits out a rating, so when Oncologists, before they even walk in to see you, they probably look at that and go, oh yeah, this person is not doing [well] and so it’s standardizing (F03).  I think it should only be one of the things that the psychiatrist…I mean they use different tools, right. Obviously a psychiatrist uses different tools. They use their own experience. They use their own you know, this, that and the other …. Their own judgement, but I think it could factor in as one of the things to give them some idea, sure (F03).  I mean the issue is you can’t measure what you can’t see…so not understanding or not knowing what next proper right steps, I think, most parents want to do what is right and will only want to do what’s right in a measurable way? (F04).  Every year, um, eh, for my follow-up appointment, they have a questionnaire, eh, uh, my pain, my energy, my appetite…I feel that they can quickly look at that and get to the point that, um, there’s an issue (F06).  So, like, maybe it’s not always, like, relevant at times. But I, I could see how, theoretically, that would be useful to confirm (F07).  Providers  In all honesty I don’t [find that weekly measurements on certain scales provides helpful information] (S01).  I think it’s about clinical care but it has a huge research component that can then loop back and inform further clinical care. You can identify gaps in service when everybody’s doing the same service to say oh geez like look at this proponent of people that need something else what is that something else and we can build more research (C02).  I didn’t see how it was informing care. I heard that you know they are collecting data. I’m assuming you know doing the MSE or the risk assessment or the safety and comfort plan like I don’t know who ever looks at this stuff I guess you know (C03).  The YMRS I know it’s built into our new ICP to be honest and maybe it’s like a deficit in my practice, mania to me is a clinical entity and I make a clinical judgement. So I don’t usually find that helpful to me (S05).  Again I don’t know if I’m systematic enough so one thing I’ll say is and I am embarrassed to admit this but I don’t know if I ever looked at one of the 18 assessments. Not all of the case managers do it but I notice some of them do. I would never open that why would I do it that’s a lot of work for me (S05).  I try to [use the QIDS]. Again I think it’s often in the context of us making some sort of change so it helps me if it determines if it helps or not (S05).  I don’t rely on the scales at all I have to say. It’s completely clinical like you know (S08).  I think they're under underutilized, just broadly speaking. I think personally I'd like to, um, myself become more comfortable using them and using the information to inform my decisions. I think in psychiatry we rely a lot on gut feeling, and kind of overall clinical assessment, but it would be nice to use numbers (S12). |
| Theme 3: Equity vs flexibility | Patients  If someone is depressed they are just going to like, I’m depressed and they are going to circle all the 0’s, and you know I could be really manic and I could be like well I am such a go-getter, well I am just a positive person and just circle all of the 10s (P08).  I think that it’s good to have this to maybe get like a vague gauge of how the client is doing at that time, but in terms of basing medication just on the scale and cognitive ability at that moment or even across time, I think that the standardized questions, I don’t think that they would suffice. I think that is very much to the doctor’s individual interpretation of what the patient is going through and I think that the doctor would have to make the call based on their own analysis as opposed to what is standardized. So I think it is good to have it like as a nice evaluation (P11).  I think again it would have to be down to the doctor to again to gauge how the patient is feeling, whether to let it all out first and say okay thank you sorry we have and take a bit of time at the end to just ask these questions quickly just to get an idea (P11).  Like they ask me questions about if I feel that I am followed or am I watching the Television and I feel that that person is also persecuting me? So, like I don’t feel that in any way, and I am glad that they ask me those questions, but I don’t have that anymore. Because sometimes you know – sometimes I feel like what bothers me is my gaining weight and I wish they could give me a solution about that (P13).  Families  I think the challenge is going to be how the person that has to fill the questionnaire is, what shape they are in at the time? Right, because based on the things that I have learnt coming here to Family Connections and learning about psychosis and what not, it’s where is the person you know where do they fit at the time, like are they stable yet, like how close are they to stable? Like are you dealing with someone that right now is refusing care or … so I would say it comes down to I think the doctor in my opinion would have to make a judgement call like is this person (F03).  Well I think that I can see that there is some advantage to that, that you are always asking the same questions, but what about the questions that never get asked?...You know, one size doesn’t fit all? Certainly, it’s good to have a guideline, but you should also be able to have the freedom to ask a question that isn’t on the list of questions to be asked (F05).  Providers  For me it’s a combination of symptoms the level of distress the clients are experiencing side effects with medication how they are functioning because I think for me a lot of not a lot but something important to keep in mind with any medication, medication changes is that clients will they don’t tolerate it sometimes or they just don’t want a higher dose and so that factors into treatment making treatment changes (S01).  Well like I don’t think it needs to negatively impact I think it’s how you go about it. People decline and you respectfully appreciate that I think it can be used to build rapport only from a I want to get to know you better, and these are some tools that I can do that (C02).  I guess I would disagree with [the notion that scales take away from the gestalt impression] because I don’t I mean because I don’t either trust my own skills and stuff like that I mean I think that gestalt feeling can be so variable I mean you know sometimes again you know psychiatry counter transference influences your assessment of patients so if you like or dislike a client and then that might influence your rating and so I think anything to take away that piece again I’m not confident in my gestalt my sort of I mean you have a sense of things I mean that’s how you diagnose pattern recognition but I think in terms of getting improvement maybe I should rephrase that not that I don’t trust that I like something to anchor myself (S04).  Well they present with psychosis they say all the symptoms but when you really look into it like it’s not functionally impairing no one will notice the psychotic symptoms. They come out in the times of traumatic events there’s often a history of sexual abuse or other disorder attachment and really when you talk to them like you could use distress tolerance in different kind of affective management tools to treat that and you see that like it’s very effective they get better (S05).  There needs to be some flexibility and from a logistical stand point I think that like offering the same thing and attempting the same thing with everybody is a good idea but we need to be able to know what to do if a client isn’t able to complete a scale or doesn’t want to complete a scale there needs to be a viable alternative so people aren’t floundering (C07).  I think the scales and everything else should be a guidance factor right as most psychiatry is like the DSM it’s more for guidance rather than a hard and fast you know way of diagnosing and treating people (S08).  It gives people the option you don’t have to participate in any of these or you know some week it’s not a good week and you know you just rather sit and talk we can do that it’s’ you know I don’t make it seem like it’s daunting mandatory task (C09).  Scales in the sense for clinical purposes I do for example the scales that I use are I use QUIDS quite often whenever I notice there’s any change in patients moods (C10).  And I’ve had people, usually people with more, like, the, the borderline and personality stuff who, um, might get dysregulated doing a scale (C11). |
| Theme 4: Shared decision making | Patients  The biggest criticism sometimes, because like I have to trust the doctor in terms of medication sometimes it is always that feeling can I truthfully be honest, because I know if I say certain things, they will take something away or try to add something and I am comfortable already sometimes or I want to do something slower (P01).  The only way that I think that it could help would be like look here is a 5, and then [case manager’s name] would be like why do you feel that it is a 5? (P08)  I think it has become a very small part of a sort of an introductory thing of what I want to talk about, so if I happen to be feeling low mood, suicidal at all, like it’s – some people around here are better at it. [My case manager] is great at it, that is why I say she is down to earth, like she can casually bring up the standard questions without really making it feel forced (P09).  It can be helpful like if someone doesn’t feel comfortable talking about something, I guess it can help them get a better understanding (P10).  It depends, because on some appointments I will have a lot to say and on some I will not have so many things to say and in those appointments I think it would be nice if some of these questions would be asked. Because they might spark something, you know…I think that even in appointments where a lot needs to be said from the patient, these questions should be asked because then they can tie the results from that particular question set to the situation in which the patient has given and see if there is a certain pattern across time in which results from that coincide with large situations (P11).  Families  I wouldn’t want to take it too far so that it’s not taking into account individual…There is a scale, there is a clinical standard, so when Dr. [X] said to me … I think it was [X]? “We would want him to take a mood stabilizer and an antipsychotic” and I am like, why an antipsychotic? And he said, “Because that is our standard thing.” I am like wow, that’s not really a good enough answer for me. (F02).  [My daughter] and I were talking just the other day because she just saw Dr. [X] this week, and they were debating again whether she should go from, like, 60 to 40 milligrams of the lamotrigine, I think, 'cause that – oh, no. I'm sorry. The Latuda, the one that gives her the weird night feeling. Um, and she was saying the other medication that she takes, which is lamotrigine, I'm guessing, uh, sh-, she said the average doshage, dosage is 200 and she’s only on 100. So it would be interesting. I feel like that’s interesting to someone who is trying to dose those kinds of medication for someone else if you know that, like, this person feels this way generally and has taken such a small dosage, maybe that’s appropriate for someone else similar to [my daughter] (F07).  Providers  What I like is the discussions that we have around that I have with the case managers around certain scales like the psychosocial functioning scales. The BCAS is one that we use it’s like a screener for cognitive functioning and we have a really long wait time to see our neurocognitive psychologist and so sometimes the BCAS is a nice bridge that helps gives some a bit of insight into how client are doing cognitively before they can get the full assessment and so it gives us something to work with. So and I like the communication and the collaboration that comes out of having these discussions with the case managers (S01).  So the case managers often will text me saying x’s QUIDS is 27 you need to come see the person (S05).  I often wondered kind of if this provides a different presentation to a person and us sitting there having a conversation you know or whether if we had you know mental status check list with us all the time and we just constantly we recorded that on a piece of paper what impact would that have on someone as opposed to just sitting having a conversation with someone (S08).  They can be helpful. Um, I guess, uh, just even in my just clinical, you know, interview, I would use something, like, less formal just in terms of 0 to 10 how are you, how are you feeling, so I think there's role for it in able t-, being able to quantify how they, how people are doing and then kind of have an objective measure, um, like, for yourself but also to kind of show the patient in terms of okay, well, you know, last week you said this and so things are actually better even though you're not feeling that they are (S13).  The Mood and Anxiety; I constantly communicate that one, so I'll say – the psychiatrist comes in and maybe I've already been with the client for half an hour. I'll say George's anxiety is still the same level as a month ago, same number, and depression is the same (C14). |
| P = patient; F = family; S = psychiatrist; C = case manager | |
