# Supplementary material for: Patient, family and provider views of measurement-based care in an early-psychosis intervention programme
Source: BJPsych Open. 2021 Sep 17;7(5):e171. doi: 10.1192/bjo.2021.1005 (PMC8485347; doi:10.1192/bjo.2021.1005)
Supplement: Supplementary file 1 [file bjosup.zip › S205647242101005Xsup002.docx]

## Supplementary Appendix 1: Qualitative Interview Guides

CLIENT VERSION

This interview guide was adapted from a template created by Dr. A. Ka Tat Tsang (Tsang, 2008).

1. **Venue**: Wherever it is convenient for the participant, and allows sufficient privacy. Interviews will be conducted in private offices at the Centre for Addiction and Mental Health. The location and time will be discussed prior to the meeting.
2. **Duration**: As long as it takes for the participants to complete their stories, although we try not to go over an hour and a half. If the participant is tired, let him or her take a break. Use your discretion if it is better to go back a second time to continue the interview.
3. **Procedures**:
   1. **Set Up**
      1. Introduce yourself and the purpose of the interview, e.g., “I am a researcher working on a study of people who are receiving care at CAMH in the first episode psychosis program. The purpose of our research study is to explore your experiences in this program and your thoughts about your care.”
      2. Explain the key content in the consent form (e.g., confidentiality and anonymity, the participant’s right to withdraw and to delete data).
      3. Explain the need for audio-recording and obtain approval from the participant. [Remember to bring your recorder and to check it for proper functioning, including sufficient battery life, memory space for recording]
      4. Obtain written consent. If the participant can't read or write, seek the participant's approval to start recording, read out the consent form and provide explanation if necessary, then obtain the participant's verbal consent, make sure that you record the whole process
   2. **Open Exploration**
      1. Start the conversation with a brief prompt, e.g., you may repeat the purpose of the research and invite the participant to share his/her experience, the following are examples of what you may want to say to the participant:

Thank you for giving us the time to do this interview with you. The main purpose of this interview is to understand your thoughts and feelings about your care at CAMH. We are most interested in your personal experience.

You can start with whatever you want to talk about first (if participants asked what they should start with).

- - 1. the main purpose of this part of the interview is to allow the participants to express themselves as freely as possible, this can be achieved by keeping in mind that:
       1. The participant decides what is important to him/her, so let them talk about whatever they want to as much as possible. That means we DO NOT control the agenda rigidly, but try to allow maximum narrative space. You may also want to make sure that you do not interrupt the participant or cut her/him off.
       2. Each individual has his/her own idea of what is relevant to the research question. You should let them talk even though you may find what he/she says is irrelevant, unless the speech is obviously cyclical or incoherent. You may, however, repeat the research question at times to remind.
       3. Respect the participant’s language by using their expression and their wordings as closely as possible, this will avoid unnecessary (mis)interpretation and narrative conditioning on our part.
       4. Use more prompts and invitations, and use less questions; e.g. invite them to elaborate on or explain about, or give examples for a topic or an experience that they have mentioned. A question-and-answer format tends to put the participant in a passive mode, and severely compromises the opportunity for the participant to volunteer information which is not on your list of questions, therefore defeating the very purpose of ethnographic or discovery-oriented interviewing. If you need to ask questions, ask open-ended and not close-ended questions. Ask specific questions only when you have collected enough information from a topic and need to know the specific details.
       5. Summarize what the participant has said would let him/her know that you've been listening, and help to build a good rapport. This is also helpful when you want to shift the conversation to another topic - make a summary first and smoothly change the topic. Try to be brief with summaries, for long summaries might turn people off.
       6. The purpose of this interview is to explore and discover, **NOT** to solve problems, provide therapy/counseling, or offer help. If you think the participant is not receiving the service he/she needs, you can make necessary referrals after the completion of the interview.
       7. Pay attention to “free information” (content not requires by your question or request, given to you freely): The participant offers as he/she responds to your prompts and questions, these are often things that the participant want to talk more about
    2. Please try to jot detail notes during the interview, this will help you to keep track of what has been said and to make summary. Please also note down your impressions, and the participant's non-verbal behaviors whenever possible. These notes can be especially valuable in the unlikely event of recording failure.
    3. When you think the open exploration part has been completed, try to summarize the main points of the conversation and ask the participant if he/she has any thing more to add. If not, thank him/her for the sharing. Then prepare them for the structured exploration part by saying something like, “In the remaining time, I am going to ask you some further questions.”
  1. **Structured INQUIRY**
     1. The purpose of structured inquiry is to focus on specific areas or issues we are interested in, but have not been addressed by the participant in the Open Exploration section. It is hoped that by this time, you would have established a good relationship with the participant and he/she might be more ready to talk about these topics
     2. Before we ask the questions, note if any of them had already been answered during the Open Exploration. Ask only those that have not been addressed. Asking the question again will make the participant feel that we have not been paying attention and listening carefully.

**Topics for exploration**

1. Tell me a few things about you and your life
2. Tell me about the people who are most important to you
3. How did you come to be receiving care at CAMH?
4. Tell me what life was like for you before you started coming here
5. When did you first experience with this program? How did you feel before you came? What did you expect?
6. Could you describe the events of your first appointment?
7. Can you tell me about the people involved in your care? Can you tell me about the different parts of your treatment?
8. Do you take medication? Do you remember who talked to you about medication? What were those conversations like?
9. When you come here you are often asked to complete scales. What is this process like for you? What are your thoughts on the scales?
10. Is your family involved in your care? Who is involved? Can you give me an example of a time it helped to have your family involved? Or a time it didn’t help?
11. Have your views about getting treatment changed over time?
12. Can you think of a moment where you felt happy, sad or mad during your care?
13. In a perfect world, what would you like to change about this program?
14. Are there any things that make it hard to come to your appointments? What would help with that?
15. How easy is it to understand information about your diagnosis or your treatment? How do you ask for help? What are things people do that help you understand? What do you wish your care team would know? What would you like to teach them?

*If the participant introduces the following concepts, the interviewer will ask open- ended clarifying and exploratory questions pertaining to that topic.*

1. Negotiating illness and identity
2. Navigating power imbalances
3. Experiences of medication
4. Family involvement in care and relationship stressors
5. Feeling “medicalized” or like “just a number”
6. Structural barriers to attend appointments
7. Autonomy vs. dependence
8. Ways of communicating distress
9. Recovery and resilience
10. Coping strategies
11. Self as expert in care
12. Experiences of help-seeking

*Ways in which to ask follow up questions about sensitizing topics (probes and clarification)*

1. Can you tell me more about that (person, event)?
2. Can you give me a specific example?
3. Can you explain your answer?
4. In what way?
5. How did you understand that?
6. What does that mean to you?

*Wrap up questions*

1. Do you have anything to add?
2. Is there anything I should have asked?
3. How did the interview feel for you?
4. Is there anything that surprised you?
5. How are you feeling now?

FAMILY VERSION

This interview guide was adapted from a template created by Dr. A. Ka Tat Tsang (Tsang, 2008).

1. **Venue**: Wherever it is convenient for the participant, and allows sufficient privacy. Interviews will be conducted in private offices at the Centre for Addiction and Mental Health. The location and time will be discussed prior to the meeting.
2. **Duration**: As long as it takes for the participants to complete their stories, although we try not to go over an hour and a half. If the participant is tired, let him or her take a break. Use your discretion if it is better to go back a second time to continue the interview.
3. **Procedures**:
   1. **Set Up**
      1. Introduce yourself and the purpose of the interview, e.g., “I am a researcher working on a study of people whose family members are receiving care at CAMH in the first episode psychosis program. The purpose of our research study is to explore your experiences in this program and your thoughts about your care.”
      2. Explain the key content in the consent form (e.g., confidentiality and anonymity, the participant’s right to withdraw and to delete data).
      3. Explain the need for audio-recording and obtain approval from the participant. [Remember to bring your recorder and to check it for proper functioning, including sufficient battery life, memory space for recording]
      4. Obtain written consent. If the participant can't read or write, seek the participant's approval to start recording, read out the consent form and provide explanation if necessary, then obtain the participant's verbal consent, make sure that you record the whole process
   2. **Open Exploration**
      1. Start the conversation with a brief prompt, e.g., you may repeat the purpose of the research and invite the participant to share his/her experience, the following are examples of what you may want to say to the participant:

Thank you for giving us the time to do this interview with you. The main purpose of this interview is to understand your thoughts and feelings about your family member’s care at CAMH. We are most interested in your personal experience.

You can start with whatever you want to talk about first (if participants asked what they should start with).

- - 1. the main purpose of this part of the interview is to allow the participants to express themselves as freely as possible, this can be achieved by keeping in mind that:
       1. The participant decides what is important to him/her, so let them talk about whatever they want to as much as possible. That means we DO NOT control the agenda rigidly, but try to allow maximum narrative space. You may also want to make sure that you do not interrupt the participant or cut her/him off.
       2. Each individual has his/her own idea of what is relevant to the research question. You should let them talk even though you may find what he/she says is irrelevant, unless the speech is obviously cyclical or incoherent. You may, however, repeat the research question at times to remind.
       3. Respect the participant’s language by using their expression and their wordings as closely as possible, this will avoid unnecessary (mis)interpretation and narrative conditioning on our part.
       4. Use more prompts and invitations, and use less questions; e.g. invite them to elaborate on or explain about, or give examples for a topic or an experience that they have mentioned. A question-and-answer format tends to put the participant in a passive mode, and severely compromises the opportunity for the participant to volunteer information which is not on your list of questions, therefore defeating the very purpose of ethnographic or discovery-oriented interviewing. If you need to ask questions, ask open-ended and not close-ended questions. Ask specific questions only when you have collected enough information from a topic and need to know the specific details.
       5. Summarize what the participant has said would let him/her know that you've been listening, and help to build a good rapport. This is also helpful when you want to shift the conversation to another topic - make a summary first and smoothly change the topic. Try to be brief with summaries, for long summaries might turn people off.
       6. The purpose of this interview is to explore and discover, **NOT** to solve problems, provide therapy/counseling, or offer help. If you think the participant is not receiving the service he/she needs, you can make necessary referrals after the completion of the interview.
       7. Pay attention to “free information” (content not requires by your question or request, given to you freely): The participant offers as he/she responds to your prompts and questions, these are often things that the participant want to talk more about
    2. Please try to jot detail notes during the interview, this will help you to keep track of what has been said and to make summary. Please also note down your impressions, and the participant's non-verbal behaviors whenever possible. These notes can be especially valuable in the unlikely event of recording failure.
    3. When you think the open exploration part has been completed, try to summarize the main points of the conversation and ask the participant if he/she has any thing more to add. If not, thank him/her for the sharing. Then prepare them for the structured exploration part by saying something like, “In the remaining time, I am going to ask you some further questions.”
  1. **Structured INQUIRY**
     1. The purpose of structured inquiry is to focus on specific areas or issues we are interested in, but have not been addressed by the participant in the Open Exploration section. It is hoped that by this time, you would have established a good relationship with the participant and he/she might be more ready to talk about these topics
     2. Before we ask the questions, note if any of them had already been answered during the Open Exploration. Ask only those that have not been addressed. Asking the question again will make the participant feel that we have not been paying attention and listening carefully.

**Topics for exploration**

1. Tell me a few things about you and your life
2. Tell me about your loved one who is receiving care at CAMH. What were they like as a child? What are they like now? What are some things they enjoy?
3. How did they come to be receiving care at CAMH?
4. Tell me what life was like for you before you started coming here
5. When did you first experience with this program? How did you feel before you came? What did you expect?
6. Could you describe the events of the first appointment?
7. Can you tell me about the people involved in your loved one’s care? Can you tell me about the different parts of his or her treatment?
8. Do you loved one medication? Do you remember who talked to him or her about medication? What were those conversations like? Did anyone talk to you?
9. When you come here your loved one is often asked to complete scales. What is this process like for him or her? What are your thoughts on the scales?
10. What have been the challenges in being involved in care? Have there been times you felt that you were less involved than you would like to be? Have there been times you felt more involved than you would like to be?
11. Have your views about illness and treatment changed over time?
12. Can you think of a moment where you felt happy, sad or mad during your family member’s care?
13. In a perfect world, what would you like to change about this program?
14. Are there any things that make it hard to for your loved one to come to appointments? What would help with that?
15. How easy is it to understand information about your loved one’s care or treatment? How is information provided? Do you have any advice? What do you want to teach the care team?

*If the participant introduces the following concepts, the interviewer will ask open- ended clarifying and exploratory questions pertaining to that topic.*

1. Confidentiality vs. Involvement
2. Conflicting priorities of care
3. Caregiver burnout
4. Difficulties communicating with team
5. Positive or negative personal factors in care providers
6. Positive or negative aspects of the care
7. Psychoeducation as process
8. Client autonomy vs. family desires
9. Financial stress
10. Transitioning through developmental life stages

*Ways in which to ask follow up questions about sensitizing topics (probes and clarification)*

1. Can you tell me more about that (person, event)?
2. Can you give me a specific example?
3. Can you explain your answer?
4. In what way?
5. How did you understand that?
6. What does that mean to you?

*Wrap up questions*

1. Do you have anything to add?
2. Is there anything I should have asked?
3. How did the interview feel for you?
4. Is there anything that surprised you?
5. How are you feeling now?

CARE PROVIDER VERSION

This interview guide was adapted from a template created by Dr. A. Ka Tat Tsang (Tsang, 2008).

1. **Venue**: Wherever it is convenient for the participant, and allows sufficient privacy. Interviews will be conducted in private offices at the Centre for Addiction and Mental Health. The location and time will be discussed prior to the meeting.
2. **Duration**: As long as it takes for the participants to complete their stories, although we try not to go over an hour and a half. If the participant is tired, let him or her take a break. Use your discretion if it is better to go back a second time to continue the interview.
3. **Procedures**:
   1. **Set Up**
      1. Introduce yourself and the purpose of the interview, e.g., “I am a researcher working on a study of people whose family members are receiving care at CAMH in the first episode psychosis program. The purpose of our research study is to explore your experiences in this program and your thoughts about your care.”
      2. Explain the key content in the consent form (e.g., confidentiality and anonymity, the participant’s right to withdraw and to delete data).
      3. Explain the need for audio-recording and obtain approval from the participant. [Remember to bring your recorder and to check it for proper functioning, including sufficient battery life, memory space for recording]
      4. Obtain written consent. If the participant can't read or write, seek the participant's approval to start recording, read out the consent form and provide explanation if necessary, then obtain the participant's verbal consent, make sure that you record the whole process
   2. **Open Exploration**
      1. Start the conversation with a brief prompt, e.g., you may repeat the purpose of the research and invite the participant to share his/her experience, the following are examples of what you may want to say to the participant:

Thank you for giving us the time to do this interview with you. The main purpose of this interview is to understand your thoughts and feelings about your family member’s care at CAMH. We are most interested in your personal experience.

You can start with whatever you want to talk about first (if participants asked what they should start with).

- - 1. the main purpose of this part of the interview is to allow the participants to express themselves as freely as possible, this can be achieved by keeping in mind that:
       1. The participant decides what is important to him/her, so let them talk about whatever they want to as much as possible. That means we DO NOT control the agenda rigidly, but try to allow maximum narrative space. You may also want to make sure that you do not interrupt the participant or cut her/him off.
       2. Each individual has his/her own idea of what is relevant to the research question. You should let them talk even though you may find what he/she says is irrelevant, unless the speech is obviously cyclical or incoherent. You may, however, repeat the research question at times to remind.
       3. Respect the participant’s language by using their expression and their wordings as closely as possible, this will avoid unnecessary (mis)interpretation and narrative conditioning on our part.
       4. Use more prompts and invitations, and use less questions; e.g. invite them to elaborate on or explain about, or give examples for a topic or an experience that they have mentioned. A question-and-answer format tends to put the participant in a passive mode, and severely compromises the opportunity for the participant to volunteer information which is not on your list of questions, therefore defeating the very purpose of ethnographic or discovery-oriented interviewing. If you need to ask questions, ask open-ended and not close-ended questions. Ask specific questions only when you have collected enough information from a topic and need to know the specific details.
       5. Summarize what the participant has said would let him/her know that you've been listening, and help to build a good rapport. This is also helpful when you want to shift the conversation to another topic - make a summary first and smoothly change the topic. Try to be brief with summaries, for long summaries might turn people off.
       6. The purpose of this interview is to explore and discover, **NOT** to solve problems, provide therapy/counseling, or offer help. If you think the participant is not receiving the service he/she needs, you can make necessary referrals after the completion of the interview.
       7. Pay attention to “free information” (content not requires by your question or request, given to you freely): The participant offers as he/she responds to your prompts and questions, these are often things that the participant want to talk more about
    2. Please try to jot detail notes during the interview, this will help you to keep track of what has been said and to make summary. Please also note down your impressions, and the participant's non-verbal behaviors whenever possible. These notes can be especially valuable in the unlikely event of recording failure.
    3. When you think the open exploration part has been completed, try to summarize the main points of the conversation and ask the participant if he/she has any thing more to add. If not, thank him/her for the sharing. Then prepare them for the structured exploration part by saying something like, “In the remaining time, I am going to ask you some further questions.”
  1. **Structured INQUIRY**
     1. The purpose of structured inquiry is to focus on specific areas or issues we are interested in, but have not been addressed by the participant in the Open Exploration section. It is hoped that by this time, you would have established a good relationship with the participant and he/she might be more ready to talk about these topics
     2. Before we ask the questions, note if any of them had already been answered during the Open Exploration. Ask only those that have not been addressed. Asking the question again will make the participant feel that we have not been paying attention and listening carefully.

**Topics for exploration**

1. Can you tell me a little bit about yourself? Your background? Your role within this program?
2. What does a typical day look like for you? A recent day?
3. What drew you to working within this program?
4. Tell me about your experience of the Integrated Care Pathway for First Episode Psychosis. When did you first hear about it? How was it developed? What were your initial thoughts?
5. Take me through your typical workflow for a new client before and after this program
6. What do you think is working? What isn’t working?
7. Tell me about your experiences using scales
8. Tell me about your experiences of the medication management protocol
9. What are some unintended consequences of the ICP, positive or negative?
10. How do you think patients respond to this type of care?
11. How do you think families respond to this type of care?

*Sensitizing concepts*

1. Top-down vs. Collaborative decision making
2. Rapport building
3. Time management
4. Feeling “heard”
5. Unintended consequences
6. Clinician identity
7. Patient related factors

*Ways in which to ask follow up questions about sensitizing topics (probes and clarification)*

1. Can you tell me more about that (person, event)?
2. Can you give me a specific example?
3. Can you explain your answer?
4. In what way?
5. How did you understand that?
6. What does that mean to you?

*Wrap up questions*

1. Do you have anything to add?
2. Is there anything I should have asked?
3. How did the interview feel for you?
4. Is there anything that surprised you?
5. How are you feeling now?
