# Supplementary material for: Patient, family and provider views of measurement-based care in an early-psychosis intervention programme
Source: BJPsych Open. 2021 Sep 17;7(5):e171. doi: 10.1192/bjo.2021.1005 (PMC8485347; doi:10.1192/bjo.2021.1005)
Supplement: Supplementary file 1 [file bjosup.zip › S205647242101005Xsup003.docx]

Supplementary Appendix 2: Reflexivity Statements

Ari Cuperfain is a resident in psychiatry at the University of Toronto. His past research has focused on the pharmacogenetics of psychotropic medication side effects. He has also participated in qualitative studies involving patients with limited English proficiency. He is supervised by Dr. Juveria Zaheer, who is a general psychiatrist experienced in qualitative research and methodology

Katrina Hui is a resident in psychiatry at the University of Toronto. Her research has focused on the addictions, bioethics, implicit biases. She is supervised by Dr. Juveria Zaheer, who is a general psychiatrist experienced in qualitative research and methodology.

Suze Berkhout was a psychiatry resident at the time of the study, carrying out ethnographic research in the same clinic setting. She additionally worked clinically in both inpatient and outpatient first episode psychosis settings, at times when she was not involved in research. She brings to her research praxis theoretical and political commitments based in intersectional feminist philosophy and science and technology studies (STS).

George Foussias is a clinician scientist and psychiatrist at CAMH, and is the Medical Head at the Slaight Centre. Of relevance to this study, he has both clinical and research expertise in schizophrenia and early psychosis.

David Gratzer is a psychiatrist at CAMH who is completing a fellowship with the VA in quality improvement. He does not work clinically within the Slaight program. He was involved in the writing of this paper, and in the literature review.

Sean Kidd is a Clinical Psychologist who has been an Investigator affiliated with the SCEIS for 5 years. His experience includes qualitative, quantitative and mixed methods designs and his work has concentrated on interventions for homeless youth populations, peer support, and cognitive interventions for psychosis populations.

Nicole Kozloff is a child and adolescent psychiatrist and has worked as a clinician-scientist in SCEIS for 3 years. She has training in health services research methods and studies mental health service use among young people with serious mental illness, with a focus on service engagement in early psychosis intervention.

Paul Kurdyak a psychiatrist at CAMH with expertise in population mental health and schizophrenia outcomes research. He does not work clinically within the Slaight program. He interpreted the data and contributed to the drafting of the manuscript.

Brandon Linaksita was both the Research Assistant for The Slaight Family Centre for Youth in Transition and the Research Analyst for the current study. His current highest academic degree is BScH.

Dielle Miranda is a research program manager at the Slaight Centre, CAMH. She worked closely with Dr. Zaheer to oversee the successful recruitment and coordination of this study. She did not have access to any of the anonymized interview transcripts to protect confidentiality of the respondents.

Sophie Soklaridis is a senior scientist and has collaborated with SCEIS for 3 years. She has training in qualitative research methods and a particular interest in the inclusion of patients and family in mental health education research.

Aristotle Voineskos is a psychiatrist at CAMH and at the time of the study, the Director of the Slaight Centre. Of relevance to this study, he has both clinical and research expertise in schizophrenia and early psychosis.

Juveria Zaheer is a psychiatrist at CAMH with expertise in qualitative research methods and qualitative program evaluation. She does not work clinically within the Slaight program. She led the study design, conducted the majority of the qualitative interviews, and led the analysis.
